# Retraction: Diagnosis and Treatment Modalities for Osteomyelitis

**DOI:** 10.7759/cureus.r144

**Published:** 2024-07-03

**Authors:** Yash Jha, Kirti Chaudhary

**Affiliations:** 1 Orthopedics, Jawaharlal Nehru Medical College, Datta Meghe Institute of Medical Sciences, Wardha, IND; 2 Anatomy, Jawaharlal Nehru Medical College, Datta Meghe Institute of Medical Sciences, Wardha, IND

This article has been retracted by the Editors-in-Chief due to plagiarism. Figures 1, 2, 4, 5, 6, and 7 have been intentionally misattributed to the authors as all six include a note that they were taken by first author Yash Jha. This is false, as the images originated in the following sources and were taken by the authors without credit or permission:

1 and 2: Sambri, Andrea, et al. "Bone and joint infections: The role of imaging in tailoring diagnosis to improve patients’ care." Journal of Personalized Medicine 11.12 (2021): 1317. https://doi.org/10.3390/jpm11121317

Figure 4: Desimpel, Julie, Magdalena Posadzy, and Filip Vanhoenacker. "The many faces of osteomyelitis: a pictorial review." Journal of the Belgian Society of Radiology 101.1 (2017). https://doi.org/10.5334/jbr-btr.1300

Figure 5: Ibrahim D, Osteomyelitis - tibia. Case study, Radiopaedia.org (Accessed on 21 Jun 2024) https://doi.org/10.53347/rID-72866

Figure 6: Gaillard F, Osteomyelitis - proximal tibia. Case study, Radiopaedia.org (Accessed on 21 Jun 2024) https://doi.org/10.53347/rID-7651

Figure 7: Jones J, Osteomyelitis with sequestrum. Case study, Radiopaedia.org (Accessed on 21 Jun 2024) https://doi.org/10.53347/rID-62732

